# Determinants of adult vaccination at inner-city health centers: A descriptive study

**DOI:** 10.1186/1471-2296-7-2

**Published:** 2006-01-10

**Authors:** Mary Patricia Nowalk, Richard K Zimmerman, Melissa Tabbarah, Mahlon Raymund, Ilene K Jewell

**Affiliations:** 1 Department of Family Medicine and Clinical Epidemiology, University of Pittsburgh School of Medicine, 3518 Fifth Avenue, Pittsburgh, PA 15261 USA; 2 Department of Behavioral and Community Health Sciences, University of Pittsburgh Graduate School of Public Health, 130 DeSoto Street, Pittsburgh, PA 15261 USA

## Abstract

**Background:**

Pneumococcal polysaccharide vaccination rates among adults 65 years and older or less than 65 years with high risk medical conditions are still below *Healthy People 2010 *recommended levels of 90%. This study was designed to: 1) assess self-reported pneumococcal vaccination rates following health center level interventions to increase adult vaccination rates; and 2) determine factors associated with vaccination.

**Methods:**

Tailored interventions to increase immunizations were implemented at two inner-city health centers. We surveyed 375 patients 50 years of age and older. Multivariate logistic regression examines the predictors of 1) self-reported pneumococcal vaccination and 2) combined self-reported influenza and pneumococcal vaccination. Both of these models were stratified by age group (50–64 years and 65 years and older).

**Results:**

Pneumococcal vaccination rates were 45% by self-report, 55% by medical record review, 69% for patients 65 years old and older, 32% for patients 50–64 years; they did not differ by race. Receipt of the previous season's influenza vaccine was significantly related to pneumococcal vaccination among both younger and older patients. Receiving both the pneumococcal vaccine and the most recent influenza vaccine compared with receiving neither, among younger patients was related to unemployment, more frequent physician visits, and belief that those who do not receive the flu shot are more susceptible to the flu. For older patients, receipt of both vaccines was related to nonsmoking status, believing that friends/family think the patient should be vaccinated, seeing posters advertising flu shot clinics, and belief that those who do not receive the flu shot are more susceptible to the flu.

**Conclusion:**

Our findings suggest that improving overall pneumococcal vaccination rates among eligible adults, has the potential to eliminate racial disparities. Interventions delivering vaccination messages specific to older and younger adult groups may be the best strategy for improving adult vaccination rates.

## Background

Pneumonia and influenza together are the fifth leading cause of death in the elderly (people 65 years old and older) and the seventh leading cause of death among all ages in the United States [1]. For this reason, pneumococcal polysaccharide vaccination is recommended for all adults 65 years old and older and those 64 years and younger who have chronic pulmonary, cardiac, renal, or liver disease or are immunocompromised. Yet in 2002, national vaccination rates were 55% for adults 65 years old and older and 8.4% for all adults 18 to 64 years [2]. Clearly, efforts to increase pneumococcal vaccination rates have fallen short of *Healthy People 2010 *goals of 90% [3]. In a previous study, we found low overall pneumococcal vaccination rates of 57% [4] and significant (11%) racial disparity in self-reported pneumococcal vaccination rates in patients ≥65 years old, at inner-city health centers [5].

The most successful interventions to increase vaccination rates are those that include patient-, provider-, and system-oriented strategies [6]. The Task Force on Community Preventive Services strongly recommends patient and provider reminders, assessment and feedback of performance, and standing orders, among other tactics to increase vaccination rates [6,7]. However, a one-size-fits-all approach is unlikely to be successful because the nature of primary care offices varies depending upon their organizational structure, setting, goals, culture, physician philosophies, patient demographics and patient medical problems [8]. Interventions shown to be successful in suburban settings may not transfer similarly to inner-city practices. Several authors have suggested tailoring preventive services interventions to match the unique characteristics of each primary care office as a means of increasing the likelihood of success [8-11].

Two inner-city health centers serving large minority populations and in which racial disparities in vaccination rates had been previously reported, [5] developed and implemented strategies to improve adult vaccination rates and reduce racial disparities. A sample of patients was surveyed regarding vaccination status and facilitators of and barriers to vaccination. The purpose of this study was to report pneumococcal vaccination rates following that effort, as well as factors related to receipt of adult vaccines.

## Methods

### Site descriptions

The intervention sites were faith-based neighborhood health centers that serve the disadvantaged in inner-city neighborhoods. They are located in low-income urban neighborhoods, have similar missions, and have approximately the same patient demographic distribution. One is approximately twice as large as the other, with two offices and has a greater proportion of uninsured and medical assistance patients.

### Interventions

Intervention staff met with the leadership of each health center to discuss their interest in participating in the study and to present a menu of techniques known to be effective in increasing immunization rates [6]. These interventions included patient-oriented strategies such as mailed reminders, posters in waiting and exam rooms and in the community; provider-oriented strategies such as chart reminders or prompts; and system-oriented strategies such as standing orders for nursing staff to vaccinate without a written order from the physician, walk-in flu shot clinics and the ability to provide vaccine free of charge to uninsured patients. Educational sessions for clinical care staff were conducted to inform them about current immunization recommendations, the purpose and significance of the interventions and to answer any questions. Health center personnel were encouraged to offer pneumococcal polysaccharide vaccine at the same time as patients were being given influenza vaccine. Intervention staff also provided immunization posters, developed mailers, assisted with preparing the mailings and helped procure free influenza vaccine supplies.

### Survey sample

Random samples of patients from each health center, generated from billing lists, were invited to participate in a telephone survey following the 2001–02 influenza season. To be eligible, patients were at least 50 years old (as of October 1, 2000) and had been seen at one of the health centers in the last year. Patients were excluded if they were deaf, homeless, had severe psychosis or dementia, resided in a nursing home, or outside the Pittsburgh metropolitan area.

### Survey development

The questionnaire was based on the Triandis model for consumer decision-making from the Theory of Reasoned Action which includes facilitating conditions (e.g., ease of getting to a place for immunizations); and behavioral intention, consisting of attitude about the activity (e.g., getting an influenza or pneumococcal vaccine is wise); social influences (e.g., doctor or family member recommends the vaccine); and the value of the consequences of the activity (e.g., the vaccine prevents disease). The model predicts a variety of behaviors well [12-15], including exercise [14] and birth control/fertility behavior [13]. It has been used in different cultural and economic situations [13] and, as used for influenza immunization, has been shown to be internally consistent and externally valid (Cronbach's alpha 0.79 to 0.91) [12].

### Survey

The questionnaire was designed by a multi-disciplinary team using an iterative process, to assess vaccination status, impact of interventions, and patients' attitudes and beliefs about adult immunizations. The final version contained approximately 57 questions, depending upon skip pattern, including multiple choice items and Likert scale items.

### Survey procedures

A personalized introductory letter and a letter from the patient's health center endorsing the project and encouraging participation were sent to each of the sampled patients. A $10 honorarium was offered for completing the survey. Trained interviewers conducted the telephone interviews between August and October 2002 using computer-assisted telephone interviewing (CATI). Use of CATI allowed for direct data entry during the interviews, directed the sequence of questioning, prevented skipped questions through automated skip patterns, and blocked illogical or out of range values. At the end of the interview, all participants were offered the opportunity to participate in the medical record review. A subset of individuals signed a consent form agreeing to medical record review and received an additional $10 honorarium.

### Medical record review

For patients who provided consent, electronic medical records and paper charts were examined for receipt of pneumococcal polysaccharide vaccine. Data were entered directly into an electronic spreadsheet and summarized. Pneumococcal vaccination from self-report and medical records was compared using the medical record as the gold standard. Sensitivity, specificity, positive predictive value and negative predictive value were calculated.

### Statistical analysis

We calculated weights based on the achieved sample to account for different sampling fractions and stratification by age group and site. Chi-squared tests were weighted and used to compare vaccinated and unvaccinated patients stratified by age group (50–64 years old and 65 years old and older). Frequency data are reported as weighted percentages only (reported sample sizes are unweighted). Logistic regression analyses were then performed including all independent variables that were associated in bivariate analyses with the respective dependent variable at *P*≤0.10 and *a priori*, the variable site. Analyses were performed for two outcome variables: 1) ever receiving the pneumococcal vaccine; and 2) ever receiving the pneumococcal vaccine and receiving the most recent influenza vaccine. For the outcome variable, "ever receiving pneumococcal vaccine," two variables significantly associated in bivariate analyses, i.e., whether a regular physician recommended the pneumococcal vaccine and whether the patient received the influenza vaccine in 2001–2002 were correlated (r = 0.3, *P *< 0.001). Additionally, only 15% (n = 8) of patients who reported receiving physician recommendation for pneumococcal vaccine, had not received the vaccine. For these reasons, we chose to exclude the recommendation of the physician in these models. Furthermore, due to the high correlation (| r | > 0.3) of the Triandis variables specific to influenza and pneumonia, we were unable to combine these variables in any meaningful way. As such, we selected *a priori *the Triandis variables specific to influenza for inclusion in the multivariate analyses. None of the interactions between site and vaccination status was significant. Statistical significance was set at *P*≤0.05 and all statistical analyses were performed using SAS software (SAS Inc, Cary, North Carolina).

This project was approved by the Institutional Review Board of the University of Pittsburgh.

## Results

### Response rate

A sample of 707 patients was drawn from the health centers, of whom 59 were ineligible. Of the remaining 648, 154 could not be reached and 119 refused, leaving 375 who completed the interview, for a response rate of 58% and a refusal rate of 18%.

### Description of respondents and vaccination rates

Weighted percentages indicated that approximately two-thirds of respondents were females and 50–64 years old; less than one-half had more than a high school education; most had low to modest income levels and approximately half of the participants were black (Table 1). One third rated their health as fair or poor, while most visited their doctors three or more times per year, were nonsmokers, regularly used seat belts and took dietary supplements such as vitamin and mineral supplements. Only 45% reported ever receiving pneumococcal vaccine. In the subset of individuals for whom medical record review was available, the overall pneumococcal vaccination rate was 55.1%. When self-reported vaccination was compared with medical records, there was a high degree of correlation with sensitivity of 0.80, specificity of 0.82, positive predictive value of 0.84, negative predictive value of 0.77 and kappa of 0.61 (*P *< 0.001).

**Table 1:** Descriptive characteristics of the sample (n = 375).

**Variable**	**%**	**n**
Site		
Health Center A	48	200
Health Center B	52	175
*Demographics*		
Age		
50–64	64	185
≥65	36	190
Gender		
Female	63	241
Male	37	134
Race		
African American	47	172
Caucasian	53	187
Marital Status		
Married	31	114
Single	14	46
Widowed	26	113
Separated/divorced	30	100
Education Level		
Elementary/some high school (grades 1 to < 12)	22	93
High school graduate/vocational or technical school	39	144
Some college/college graduate	27	95
Graduate/professional school	12	42
Household Income		
<$10,000	33	117
$10,000 – 19,999	30	104
$20,000 – 39,999	18	59
$40,000 or more	19	62
Employment Status		
Unemployed	61	252
Employed part- or full-time	39	121
*Health Behaviors*		
Self-rated health		
Excellent/Very good	38	143
Good	31	120
Fair/poor	31	110
Physician visit frequency		
Every 1–2 months	25	96
3–4 times/year	36	137
Less than 2 times/year	39	139
Time since last complete physical exam		
< 1 year	73	273
1–2 years	16	59
> 2 years	11	36
Smoking status		
Current smoker	27	93
Never a smoker	31	119
Former smoker	42	163
Frequency of seatbelt use		
Always	67	253
Sometimes	23	83
Never	10	34
Use of dietary supplements		
Yes	63	236
No	37	139
Received 2001–02 influenza vaccine		
Yes	53	210
No	47	161
Ever received pneumococcal vaccine		
Yes	45	183
No	55	179

Note: All percentages are weighted and obtained using SAS; Ns are unweighted. Percentages may not add to 100% due to rounding error.

Although black participants reported lower educational and income levels, lower self-rated health and fewer were employed, they visited their physicians more frequently, had had a more recent complete physical exam and were more often smokers. Notably, there were no differences overall between blacks (49%) and whites (42%) in pneumococcal immunization status by self-report (*P *= 0.215).

Recommendations for pneumococcal vaccination differ for those older or younger than 65 years; therefore, all subsequent analyses were stratified by age group. Self-reported pneumococcal vaccination rates were 69% for those 65 years and older and 32% for those 50–64 years.

Among the 50–64 year old group, those who were unemployed, visited the doctor more frequently, had a more recent complete physical exam, self rated their health lower and received the 2001–02 influenza vaccine, were significantly more likely than their respective counterparts, to have ever received the pneumococcal vaccine (*P *< 0.05). Likewise, a positive attitude toward the pneumococcal vaccine and having had the vaccine recommended by their physician, or relatives or friends was associated with significantly higher pneumococcal vaccination rates (*P *< 0.001). Racial differences in pneumococcal vaccination rates among younger patients (39% for blacks vs. 26% for whites; *P *= 0.061) were not significant.

Among those 65 years and over, being a woman, being a nonsmoker, taking dietary supplements, having a more recent complete physical exam, and having received the 2001–02 influenza vaccine were all significantly associated with higher pneumococcal vaccination rates (*P *< 0.05). As in the younger group, a positive attitude toward pneumococcal vaccine and having received a recommendation to get the pneumococcal vaccine were significantly associated with higher pneumococcal vaccination rates (*P *< 0.001). There were no significant differences in pneumococcal vaccination rates by race in this age group either (67% for blacks vs. 70% for whites; *P *= 0.624). In logistic regression analyses (Table 2), two factors increased the likelihood of receiving pneumococcal vaccine in the 50–64 year-old group: seeing a physician more frequently and receiving the 2001–02 influenza vaccine. Among adults 65 years and older, females, as well as those who had received the 2001–02 influenza vaccine, were more likely to have received the pneumococcal vaccine.

**Table 2:** Determinants* of receipt of pneumococcal polysaccharide vaccine by age group.

**Variable**	**50–64 years^**a **^n = 180**		**≥65 years^**b **^n = 182**	
	
	**Odds ratio (95% CI)**	***P*-Value**	**Odds ratio (95% CI)**	***P*-Value**
Female (referent, male)			3.79 (1.47 – 9.74)	.006
Received the influenza vaccine in 2001–02	5.78 (2.51 – 13.30)	<.001	12.88 (5.36 – 30.95)	<.001
Last physical was < 1 year ago	2.82 (1.08 – 7.36)	.034		
Frequency of visits to physician (referent, <1 per year)				
6–12 times per year	3.39 (1.12 – 10.29)	.031		
3–4 times per year	1.82 (0.69 – 4.81)	.229		

*By logistic regression

^a^Controlling for site, race, employment status, self-rated health.

^b^Controlling for site, smoking status, use of dietary supplements, and recency of last physical exam.

Because receipt of influenza vaccine was so highly related to receipt of pneumococcal vaccine, we wished to examine the relationship between various factors and receipt of both influenza and pneumococcal vaccines, compared with receipt of neither vaccine. Bivariate analyses by age group indicated that receipt of both vaccines among patients 50–64 years was significantly associated with being unemployed, having health insurance, fair or poor self-reported health, more recent complete physical exam and more frequent visits to the physician (*P *< 0.005). For the older group, having better self-rated health, being a nonsmoker, having health insurance, more recent complete physical exam and having seen posters advertising flu shot clinics were associated with receiving both vaccines (*P *< 0.05). In bivariate analyses, most Triandis model factors were similarly associated with receipt of both vaccines as they were to receipt of pneumococcal vaccine alone, for both age groups.

Table 3 shows significant factors related to receiving both pneumococcal vaccine and the most recent influenza vaccine compared with having received neither using logistic regression. For patients 50–64 years, being employed was associated with a lower odds ratio of being vaccinated, while more frequent visits to the physician and belief that a person who does not get the flu shot will probably get the flu were associated with higher likelihood of being vaccinated against pneumococcus and receiving the most recent influenza vaccine. For patients 65 years old and older, being a nonsmoker, having seen posters advertising flu shot clinics, believing that a person who does not get the flu shot will probably get the flu, and having family or friends who feel that the patient should get the flu shot were positively associated with receiving both vaccines.

**Table 3:** Determinants* of receiving pneumococcal polysaccharide vaccine ever and most recent influenza vaccine (n = 145) compared with having received neither vaccine (n = 121)

**Variable**	**50–64 years^**a **^Odds Ratio (95% CI)**	***P*-value**	**≥65 years^**b **^Odds Ratio (95% CI)**	***P*-value**
Employed part- or full-time (referent, unemployed)	.33 (.11 – .96)	.042		
Frequency of visits to physician (referent, <1 per year)				
6–12 times per year	4.40 (1.09 – 17.78)	.038		
3–4 times per year	8.77 (2.36 – 32.62)	.001		
Smoking status (referent, current smoker)				
Never smoked			10.67 (1.67 – 68.28)	.012
Quit smoking			7.51 (1.38 – 40.84)	.020
Saw posters for flu shot clinic			5.85 (1.60 – 21.37)	.008
Believes that a person who does not get the flu shot will probably get the flu	4.67 (1.61 – 13.54)	.005	3.98 (1.01 – 15.71)	.048
My family/friends think I should get the flu shot			10.28 (2.85 – 37.00)	.001

*By logistic regression

^a^Controlling for site, race, and self-rated health, recency of last physical exam.

^b^Controlling for site, use of dietary supplements, and recency of last physical exam.

## Discussion

In an earlier study of adults over age 65 in inner-city health centers, we reported low overall immunization rates and significant racial disparities in immunization rates for both influenza and pneumococcal vaccines [4,5,16], indicating a need for interventions to reduce these disparities. After implementing tailored interventions to increase adult immunizations at two of the previously studied inner-city health centers, we found no differences in self-reported pneumococcal vaccination rates by race in adults 50 years and older. This is consistent with previous findings that tailored interventions result in increased influenza vaccinations [17]. The low overall pneumococcal polysaccharide vaccination rate of 45% however, (69% among those 65 years and older) indicates a need for considerable work if rates are to reach *Healthy People 2010 *goals of 90% [3]. We cannot attribute improvements in pneumococcal vaccination rates specifically to the interventions. However, we can conclude that whatever interventions have been undertaken, they are not perpetuating racial disparities in immunization rates that: 1) have been reported nationally (57% for whites, 32% for blacks over age 65 years) [2]; or 2) we have previously reported in these inner-city health centers (70% for whites and 59% for blacks) [5].

The differences in pneumococcal vaccination rates between younger and older adults is not unexpected, as the recommendation for those younger than age 65 years is to vaccinate only those with high risk conditions such as chronic cardiac, pulmonary, liver or kidney disease [18]. Our data indicate that among the younger adults in this study, those who saw their physicians more frequently, were unemployed and rated their health less well, were more frequently vaccinated. This suggests that those who were vaccinated may be suffering from a chronic condition that may prevent them from working, increases their contact with their physicians and makes them eligible for pneumococcal vaccination. On the other hand, among older patients, more positive health behaviors were related to pneumococcal vaccination. For instance, vaccination rates among older patients were higher among those who rated their health better, were not smokers, took dietary supplements and had a more recent physical exam. These data suggest that interventions for increasing pneumococcal vaccination should target different age groups of adults differently.

In logistic regression, the factors with the greatest impact on pneumococcal vaccination status in younger patients were having received the most recent influenza vaccine and more recent and more frequent visits to the physician. As we and others have previously reported [4,19], there is a significant relationship between physician recommendation and pneumococcal vaccination rates. More frequent office visits provide more opportunities to convey the message that the patient's physician recommends the pneumococcal vaccine. In-office strategies could serve to remind providers when the vaccine is due and to recommend the vaccine rather than just offering it. Additionally, assessment of pneumococcal vaccination status at the time of annual influenza vaccine administration is a simple and effective way of increasing pneumococcal vaccination rates.

In previous work, we and others have explored the barriers to and facilitators of adult vaccines separately [4,16,19]. This is in part because the recommendations for influenza and pneumococcal vaccines differ by vaccine and across age groups. In fact, one of the reasons cited by the National Vaccine Advisory Committee for low adult vaccination rates [20] is different target groups for different vaccines, necessitating a selective rather than universal approach. We hypothesized that there would be differences between those who report having received both vaccines (pneumococcal vaccine ever and influenza vaccine in the most recent season) and those who had received neither. We found that younger patients who received both vaccines were less likely to be employed, visited their physicians more frequently and believed in the efficacy of the influenza vaccine. Older patients who received both vaccines were more likely to: be nonsmokers, have seen posters advertising flu shot clinics and believe that family or friends think they should receive the influenza vaccine. This again suggests different influences on immunization behavior and further supports the need for targeted vaccination messages.

Although this study benefited from inclusion of an inner-city population of patients who are traditionally more difficult to reach, and a high percentage of African American respondents, the survey response rate was only moderate. Further, due to privacy issues arising from the Health Insurance Portability and Accountability Act (HIPPA) regulations, it is unknown how respondents and nonrespondents differed. Nevertheless, we found good correspondence between self-reported and medical record-derived pneumococcal vaccination status (both sensitivity and specificity ≥ 0.8).

We have reported success of tailored interventions implemented at inner-city health centers designed to increase influenza vaccination rates [17]. In this study, overall increases in pneumococcal vaccination rates reduced racial disparities observed before intervention.

## Conclusion

Our findings suggest that improving overall pneumococcal vaccination rates among eligible adults, has the potential to eliminate racial disparities. However, interventions delivering vaccination messages specific to older and younger adult groups may be the best strategy for accomplishing this task.

## Competing interests

The author(s) declare that they have no competing interests.

## Authors' contributions

MPN was the lead author, prepared the manuscript and managed editing. RKZ was responsible for study design and manuscript editing. MT performed the statistical analyses and manuscript editing. MR was responsible for data base management. IKJ managed the survey data collection. All authors read and approved the final manuscript.

## Pre-publication history

The pre-publication history for this paper can be accessed here:


